# Herbal supplements in the print media: communicating benefits and risks

**DOI:** 10.1186/s12906-019-2602-9

**Published:** 2019-08-02

**Authors:** Matthew Peacock, Mihaela Badea, Flavia Bruno, Lada Timotijevic, Martina Laccisaglia, Charo Hodgkins, Monique Raats, Bernadette Egan

**Affiliations:** 1 Food, Consumer Behaviour and Health Research Centre, School of Psychology, Faculty of Health and Medical Sciences, University of Surrey, Guildford, GU2 7XH UK; 2 Department of Fundamental, Prophylactic and Clinical Specialties, Faculty of Medicine, Transylvania University of Brasov, Bdul Eroilor Nr 29, 500039 Brasov, Romania; 3 Centre of Studies in Drug Communication, Department of Pharmacological and Biomolecular Sciences, Pharmaceutical Sciences, University Of Milan, Via Balzaretti 9, 20133 Milan, MI Italy

**Keywords:** Food supplements, Herbal remedies, Consumer information, Print media, Self-medication

## Abstract

**Background:**

The rise in use of food supplements based on botanical ingredients (herbal supplements) is depicted as part of a trend empowering consumers to manage their day-to-day health needs, which presupposes access to clear and accurate information to make effective choices. Evidence regarding herbal supplement efficacy is extremely variable so recent regulations eliminating unsubstantiated claims about potential effects leave producers able to provide very little information about their products. Medical practitioners are rarely educated about herbal supplements and most users learn about them via word-of-mouth, allowing dangerous misconceptions to thrive, chief among them the assumption that natural products are inherently safe. Print media is prolific among the information channels still able to freely discuss herbal supplements.

**Method:**

This study thematically analyses how 76 newspaper/magazine articles from the UK, Romania and Italy portray the potential risks and benefits of herbal supplements.

**Results:**

Most articles referenced both risks and benefits and were factually accurate but often lacked context and impartiality. More telling was how the risks and benefits were framed in service of a chosen narrative, the paucity of authoritative information allowing journalists leeway to recontextualise herbal supplements in ways that serviced the goals and values of their specific publications and readerships.

**Conclusion:**

Providing sufficient information to empower consumers should not be the responsibility of print media, instead an accessible source of objective information is required.

## Introduction

The food supplement market emerged from the global recession relatively unscathed [1]; in 2015 it was worth €7.2 billon to the European economy, a figure expected to grow to €7.9 billion by 2020 [2]. Food Supplements based on botanical ingredients like echinacea, valerian or gingko, comprise its second largest segment after vitamins and minerals and are typically used by around 20% of consumers in developed nations [3, 4].

Unlike vitamins and minerals, however, evidence for the effectiveness of these plant-based products is extremely variable. Manufacturers have never been obliged to provide the clinical evidence base required of prescription drugs [5] because traditionally they have been regulated more as foods, despite being marketed for remedial purposes in dose form similar to medicines.

In recent years, however, increased usage has led to calls for tighter regulation of botanical products sold within the European Union [6]. The resultant new category of Traditional Herbal Remedy allows manufacturers to market plants used for more than 30 years, including 15 in the European Union, on the basis of tradition of use [7]. Because Traditional Herbal Remedy status requires only evidence of tradition and safety, not actual efficacy, manufacturers are now mandated to remove any text implying a benefit from packaging. Although manufacturers can submit individual benefit claims for approval to the European Food Standards Authority this process has become mired in assessment difficulties, leaving thousands of claims currently on hold. As a result many manufacturers now argue they are no longer able to provide consumers with sufficient information to make informed decisions [8].

The differences underlying these two categories, coupled with the absence of any centralised authorisation procedure for botanical ingredients, has allowed a situation whereby products containing the same ingredients are frequently classified differently by the competent authorities of different EU member states [9]. In Italy, for example, most such products are classed as food supplements whilst Portugal leans towards categorising products as Traditional Herbal Medicinal Products that in many other countries would be labelled differently. The same botanical ingredients therefore often end up being used in products classed as food supplements in one state and medicines in another. This has huge implications for the ways in which information about what they are for and how to use them appropriately can be communicated to consumers [10].

Clearly, the inability of competent authorities guided by expert opinion to agree how to treat products based on botanical ingredients does not bode well for the ordinary consumer’s chances of doing so without any such guidance. Making risk-benefit decisions about botanical products is likely to become even more challenging as the botanical sector expands [11]. This paper therefore investigates the complex information environment faced by consumers navigating these choices.

The difficulty of communicating information about these products as a result of these inconsistent classifications is further demonstrated by the difficulty of finding an unambiguously agreed-upon term to refer to them in this article. After much consultation “Herbal Supplements” (HS) was chosen as a general term to describe products marketed in dose form that primarily contain botanical preparations and can be used for food supplementation, though it is fully recognised that some countries may choose to class some of these products as herbal medicines rather than supplements. For a more detailed explanation of this definition, please see endnote^1^.

### The consumer information environment

The typical HS user is older, female, educated and affluent [12, 13], supporting Raskin et al’s [11] prediction that the combination of an aging population, growing awareness of genetic risk factors and disillusionment with the medical establishment would lead to a rise in self-medication. Kessler et al. [14] depict this as part of an ongoing trend recasting the individual as the foremost expert on managing their own day-to-day health needs [15, 16].

The resultant emphasis on maintaining wellness rather than responding to illness favours the rise of supplements by reframing taking medicine as something healthy people do every day, transforming submissive patients into empowered health consumers [17, 18]. Integrating consumer frameworks into health contexts presupposes the consumer’s ability to access appropriate knowledge in order to make effective choices [19], but where are they to acquire such knowledge?

Few supplement users list conventional healthcare as a key source of HS information [20, 21], and around half who visit a physician for another reason don’t mention using supplements [21, 22]. When patients do ask doctors about supplements responses can vary greatly [23] because in many countries general practitioners are taught little about HS [24]. Instead most users first encounter supplements via recommendation by non-expert friends or relatives [22].

This helps explain worrying disparities between lay and expert beliefs about HS, including widespread assumptions that “natural” products must by definition be safe [21, 25], despite genuine risks of overdose [26] and drug interactions [27]. The belief that herbal remedies can’t hurt and may just help make some more willing to experiment with them than they would with pharmaceutical drugs [28, 29]. Worse, consumers with serious conditions have been known to risk their lives by using HS instead of conventional healthcare [30].

HS industry representatives argue that the recent regulations prevent them from plugging this knowledge gap, driving information-seekers towards unregulated and potentially unreliable sources [31, 32]. The internet, for example, is the fastest growing supplement marketplace and a popular source of HS information [20] but deciding which sites to trust can challenge even experienced web-users [33].

The prevalence of this problem for complementary medicines was summed up in a UK House of Lords report [34]:

*“There is a clear need for more effective guidance for the public as to what does or does not work and what is or is not safe in CAM. There is no central information provision for patients and healthcare practitioners; thus the media and other unregulated sources have an undue influence on opinion in the field.” (p.6).*

Indeed, mass media has played a key role in helping reframe what was a fringe activity as mainstream behaviour on the same continuum of health options as changing one’s diet or lifestyle [35]. The media’s ability to reach even those not actively seeking HS information creates the potential to convey more knowledge to more consumers than any other channel [36, 37]. Chief amongst trusted media sources is print media.

### The role of the print media

The largest demographic group of HS users - educated women over 50 – are more likely than other groups to buy magazines and newspapers [38] and less likely to be influenced by the internet [39]. Research into newspaper and magazine coverage of supplement use tends to agree that their influence is significant but inconsistent [34] and identify a worrying tendency to let the medium shape the message, potentially distorting scientific findings [40]. Ernst and Schmidt [41] find that newspapers favour simple, unidirectional messages for or against HS over attempting to do justice to an inherently complex topic, precluding the rigour necessary to help readers develop their own informed opinions [42]. Magazines, by contrast, provide more detail but often uncritically assimilate HS into their preferred style of upbeat, aspirational narrative [43], furthering what Doel and Seagrott [44] call the displacement of health into consumer culture.

These studies are now at least a decade old, a decade in which the supplement market, indeed the world of consumer information as a whole, has changed radically [45]. Simultaneously, a decade after regulatory changes attempted to bring consumers greater transparency clarity remains in short supply [46].

This paper therefore asks: what contribution are print media making to this challenging information environment? It focuses on article content, which was recommended by Entwistle and Hancock-Beaulieu [47] as more practical than trying to pick out the effect of journalism on readers’ behaviour and beliefs from a morass of other influences.

## Methods

### Study design

In order to investigate what print media are contributing to the HS information environment, researchers in Italy, Romania and the UK decided to conduct a qualitative thematic analysis of the contents of articles in the print media which focus in whole or large part on HS. It was agreed that this was the approach best suited to engaging with articles’ depictions of HS in their own terms, as this would minimise the risk of researchers imposing their own assumptions, biases and prejudices about what they expected to find.

### Choice of countries

These countries were chosen for several reasons. In part this was to provide diversity of language, culture and tradition of HS use, as well as different patterns of current use and regulation. This decision was difficult because, as Abdel-Tawab [10] points out, data comparing levels and type of use of plant-based remedies across countries without conflating them with related products such as vitamins and minerals is thin on the ground. Abdel-Tawab maintains that the only really useful study currently available in this respect is Garcia-Avarez et al’s [48] survey of consumers in six European countries (Finland, Germany, Italy, Romania, Spain and the United Kingdom).

This survey found that the highest prevalence of weighted HS usage occurred in Italy, Spain and the United Kingdom. Users in Romania, Italy and the UK were also exceptional in being much less likely to use other types of complementary or alternative medicines, which in the context of this research suggests they may well be less practiced in, and have narrower experience of, gathering and interpreting information about such topics.

At the same time these three countries were also recommended by their high variability in other important respects. The paragraphs that follow describe in more detail the factors that make each of these three countries uniquely interesting in terms of HS use.

### Italy

Italy is a uniquely interesting market for herbal supplements for several reasons, not least because it is described as the largest market for food supplements in Europe [49]. Its status as the eighth largest economy in the world with an exceptionally high life expectancy make it an ideal environment for HS use to thrive due its affluent aging population. Moreover, herbal remedies are already embedded in Italian culture to some extent, afforded a degree of legitimacy by the government and widely sold through respected outlets such as pharmacies. It stands in contrast to the other countries studied in being a founding member of the EEC in 1958 and a mainstay of the current EU, so at least in theory it has had many years to develop an effective approaching to regulating herbal products.

In practice Italy’s current approach to supplement regulation is largely guided by its partnering with France and Belgium to draw up a list of substances permissible in supplements which would then be enshrined in the laws of each country and applied consistently across borders [9]. This “BELFRIT List” was hoped to encourage further harmonisation amongst EU member states and build legal acceptance for the principle of mutual recognition across all member states [50]. Despite this, in recent years disparate patterns of regulation preferences have continued to emerge, with even Italy deciding in 2018 not to adopt the same approaches to labelling and wording of warnings as its BELFRIT partners [51].

In this and other respects Italy provides an interesting example of a country whose approach to HS regulation is to try to exert a greater degree of control. This extends to requiring compulsory registration by manufacturers of all food supplements entering the market, which are then numbered and added to a digital register of products. It has also implemented a digital notification procedure for product labelling designed both to accelerate the process and ensure they contain no information which might mislead the consumer. Italy also requires the companies themselves that manufacture, package or market products classed as food supplements to be registered and authorised by the local competent authorities [52].

### Romania

Romania provides a prime example of supplement regulation in a country with a longstanding informal tradition of herbal remedies [53] which, in contrast to Italy, only recently joined the EU.

The resulting need for a longstanding cultural staple to be rapidly regulated and brought in line with other EU nations is exacerbated by the huge growth and commercialisation of supplements in the last decade, with botanical products comprising a far larger proportion of the resulting market than vitamins and minerals in contrast to countries like the UK where vitamins and mineral supplements are much more established [54].

The reasons for this rapid growth are as much attributable to long term cultural factors as to economic growth. Professional medical care remains inaccessible to many due to geographic and economic disparities affecting provision of care in a country where natural health, care provided within communities and home-made remedies based on knowledge passed down through generations remain highly significant. This is bolstered by increasing education and advertising raising the profile of the newly commercialised supplementation sector aimed at the newly affluent and rapidly expanding middle classes. This commercialisation encompasses not just the traditionally used ingredients from indigenous manufacturers but increasingly imported formulations drawing on herbal traditions from across the world which find a ready-made market in Romania [55].

Garcia-Alvarez et al’s [48] survey paints a similar picture of informal traditions meeting newfound commercialisation but also finds other interesting respects in which Romanian HS use differs from Italy and the UK such as the unusual popularity of herbal products amongst people under 30 in a category of products more commonly associated with consumers over 50.

Romania’s approach to supplement regulation is again distinct in producing not just a positive list of approved ingredients but, like Germany and Belgium, a negative list of ingredients never to be used [56]. The complexity of regulation and classification issues facing Romanian consumers are compounded by difficulties of quality control due in part to an industry whose rate of growth is outstripping the capacity to regulate it, with neither price, provenance nor brand guaranteeing quality [55].

### The United Kingdom

Whereas Italy provides an example of a country that has been part of Europe from the beginning and Romania a relatively recent addition, the UK joined the EEC in 1973. Unlike either country, commercially available HS are a fairly new concept to mainstream UK consumers, having until recently been something of a niche market. In contrast to Romania, which offers universal healthcare in name only via what is frequently rated the worst healthcare system in Europe [57] and Italy’s mixed public-private system, the UK has a strong culture of nationalised healthcare which emphasises biomedical approaches rather than traditional remedies, as embodied in the National Health Service [58].

Against this unique background use of Complementary and Alternative medicine, often self-referred and self-funded, has grown hugely over the last 20 years [59]. The culture of supplement use which has developed in the UK appears to differ from those of the other countries in several interesting respects. Garcia-Alvarez et al’s [48] international survey of HS use found that while UK consumers were purchasing more herbal products than ever before the range of different botanical ingredients on which these products were based was much narrower than in other countries, especially Romania and Italy with their much more longstanding herbal traditions, and the number of different botanical ingredients typically incorporated in each product was likewise much smaller.

UK Healthcare institutions and the MHRA are only now beginning to fully engage with the complexities of classifying and regulating herbal products [10]. While the UK’s Medicines and Healthcare products Regulatory Agency has compiled a list of botanical ingredients and their uses, unlike Italy and Romania this list exists purely for information and lacks any legal status. The UK similarly lacks a notification process for new products entering the market. [52]

The UK is also distinct in reporting the lowest level of non-HS CAM use of complementary or alternative medicines other than HS in Garcia-Alvarez et al’s [48] survey, with 92.6% reporting not using any other kind of complementary or alternative therapy or treatment in the past year, compared to 80.8% in Romania and 74.6% in Italy).

### Procedure

Researchers in each country selected at least ten articles featuring HS from newspapers and ten from magazines. Purposeful sampling was used to ensure a range of publications including at least one magazine with a general health focus and at least one specifically addressing natural products, taking into account the spread of print articles appearing in each country to achieve deeper engagement with the print information environment.

The final sample included 35 articles from Italy, 20 from Romania and 21 from the UK, a total of 76. Further details of the articles included from each country and each individual publication, can be found in Table 1. Sampling spanned a 14-month period, allowing coverage of all seasons. Teams in participating countries agreed upon an analysis protocol which was then piloted and revised to ensure clarity and consistency.

**Table 1:** Summary of publications surveyed in each country

	Newspapaer/magazine	Title	Readership level	Readership demographics	Frequency	No. articles sampled	Other info
Italy	Newspaper	Corriere della Sera	3274000	58% male, 42% female;. Demographic: 36% Lombardy.	Daily	17	
	Newspaper	Eco di Bergamo	377000	55% male, 45% female;. Demographic: mainly Bergamo city and province	Daily	3	
	Magazine	Natural 1	11200	No Specific Demographic information available	Monthly	4	
	Magazine	OK Salute	509000	72% female, 28% male Demographic: 30% Lombardy	Monthly	11	
Italy total						35	
Romania	Newspaper	Adevarul	2nd largest in Romania.		Daily + health supplement for week end	5	National circulation. 2504943 readers of online version.
	Newspaper	Buna ziua Brasov	4th in Brasov county	45% female, 48% aged 35-54y.	Daily	5	Local circulation. 70000 visitors of online version
	Magazine	Ce se intampla doctore?	42000	General, women	Monthly	5	National circulation
	Magazine	Medicina naturista		General, women,	Monthly	5	National circulation, for those interested in natural therapies, cosmetics and medicinal plants.
Rom total						20	
UK	Newspaper	Daily Mail	4741000, 2nd largest in UK	53% female, 59% age 55+, 83% ABC1C2 demographic, 22% London area.	Daily	6	National circulation
	Newspaper	The Independent	532000, 10th largest in UK	Broad age spread, 60% male, 81% ABC1 Adult Demographic, 45% London area.	Daily	1	National circulation
	Newspaper	The Metro	3287000, 4th largest in UK	78% aged 15 to 44, predominantly commuters	Daily (Mon-Fri)	2	Free paper circulated on public transport systems of 14 UK cities, funded by advertising
	Newspaper	The Times	1565000, 6th largest in UK	58% male, 88% ABC1 Adult demographic, 31% London Area	Daily	1	National circulation
	Magazine	Healthy	160064	97% female, largely ABC1 aged 25–55	Bi-monthly	3	National circulation, published by Holland and Barrett
	Magazine	Natural Health	60000	No Specific Demographic information available, but probably broadly similar to “Healthy”	Monthly	2	National circulation, for those interesting in traditional and modern natural therapies
	Magazine	Top Sante	179000	93% female, 72% ABC1, 71% aged 35–64	Monthly	6	National circulation, features all aspects of holistic healthy, beauty and wellbeing
UK total						21	
Grand total						76	

The Articles and any related advertising were coded, the headlines translated into English, and filed as hard copies. All articles were evaluated independently by two researchers and when disagreements arose a third opinion was sought.

A number of variables were recorded for each article, documenting the most salient characteristics in a table that would allow different researchers to address the data in comparable terms, ensuring consistent interpretation across all countries. This helped researchers to consider not just what the article actually said but how it was positioned and contextualised within the publication; for example was it accompanied by pictures, how long was it and where in the newspaper or magazine did it appear (was it featured on the front page as headline news or did it appear in a pull-out section themed specifically around health or lifestyle)? The variables recorded also included the publication’s title, date of publication, the article author and its positioning/size on the page. Information on the presence of related articles and advertising was also recorded.

Team members also used a number of variables to capture the content of the articles, recording the names of plants mentioned, the main theme of the article, the purpose of the HS and any benefits or risks the article associated with the plant. They also evaluated the overall article tone as positive/negative or neutral and recorded any information sources cited.

With this initial examination of the data in mind, teams in each country then translated the data into English to allow the subsequent analysis of the text they had selected as best addressing the research interests to be undertaken by the same coders in the same language. This decision was taken after consideration of the challenges of conducting qualitative analysis on data in several different languages summarised by Squires [60].

### Data analysis

The lack of previous work on this topic mandated an exploratory method and Thematic Analysis [61] was selected as the technique best suited to a dataset of this diversity. The key reason was again the need to address each article’s presentation of HS in its own terms, allowing different narratives around supplement use to be examined without privileging any particular perspective. This approach has the advantage of using the data itself as the basis for the inductive generation and ongoing refinement of categories, facilitating a rigorous process which both retains flexibility and encourages researchers’ reflexive awareness of their own biases.

Applying this process generated initial themes which were then refined to produce overarching themes at higher levels of abstraction. The analysis was reviewed by two other researchers, revised accordingly and finalised drawing on input from the international collaborative teams.

This produced three overarching themes and ten underlying sub-themes.

## Results

The themes and sub-themes identified are listed in Table 2.

**Table 2:** Summary of themes and sub-themes from thematic analysis of the articles

Themes	Sub-themes
Risks of HS	• Health risks • Financial risks
Benefits of HS	• Health benefits • Naturalness • Taking responsibility for your own health
Evidence drawn upon to justify risks and benefits	• Scientific evidence • Expertise as evidence • Tradition of use as evidence

Almost all articles cited both benefits and risks. The analysis will consider which benefits and risks different types of publications chose to emphasise, how they justified them and what role their framing played in the overall narrative. This article uses the term “framing” to denote a choice to emphasise some elements of a topic above others [62] that “provides a way to understand an event or issue” (p.3).

Note that health-themed sections within newspapers aped the style of magazines to the extent that, with the exception of the actual labels following quotes, whenever the analysis refers to “newspapers” this means the main, news-based portion of the paper.

The extracts below were selected from articles in all three countries as exemplars of the themes drawn from analysis of the 76 articles surveyed. They draw on text agreed by the collaborators in each country to represent the themes discussed.

### Risks of HS as represented by the media

#### Health risks

This theme refers to the possibility that taking a supplement might do more harm than good, for example through overdoses or drug interactions. Articles in all countries frequently referred to health risks, albeit with huge differences in how they were presented. For example, some articles took health risks as the main thrust of their narrative.

*“Herbal remedies may be doing more harm than good because they react badly with conventional drugs, a study warns.... More than 30 per cent of shoppers are unaware of such side-effects.” UK Newspaper*

These articles varied in whether they framed the existence of risks as a shocking revelation or simply a routine aspect of ingesting unfamiliar substances.

The former framing appeared mainly in newspapers aimed at general readerships who may not be familiar with HS. The above is a more subtle example, choosing to generalise that HS “may be doing more harm than good” instead of saying, for example, that they “*can* do more harm than good” if used unwisely. Few such articles included contextual information that might moderate the risk narrative, for example the fact that any HS had to be demonstrated to be safe when used as directed in order to be marketed, preferring to offer their readers a less ambiguous message that nonetheless remains vague as to the specific nature of the risks.

By contrast, magazines in all countries, being aimed at more specific readerships than newspapers, tended to assume some existing sympathy for HS. They often framed health risks not as criticisms but as an acknowledgement that if supplements can do good they might also do harm. Typically such warnings were not the main focus of the article but followed a longer description of product benefits. Such articles also tended to be more specific about the nature of the risks:

*“While a large number of people safely enjoy the benefits of this herb, some people have reported mild gastrointestinal side effects.” UK Magazine*

*“Avoid consumption (of extracts of rosemary’s shoots) at night because can cause insomnia.” Romanian Newspaper*

The majority of articles, then, varied less in whether they mentioned risks than in how they framed them to serve their chosen narrative.

Romanian articles varied more widely in their presentation of health risks than articles from the UK. Most acknowledged the possibility of risks, though a few denied their existence entirely:

*“Capsules based on apple cider vinegar have health risks? Absolutely not. On the contrary, natural ingredients from these capsules solve body mass problems not chemically, but biologically. There are no side effects. You can eat as much as you want and still lose weight!” Romanian Newspaper*

This extract also demonstrates a tendency found in all countries to either describe or imply a dichotomy between the natural, biological and safe and the artificial, chemical and potentially toxic. Italian articles were less likely to highlight risks than UK or Romanian articles; the standard advice to consult one’s doctor before trying new supplements occasionally appeared but far more frequent were lists of side-effects that definitely would not occur.

*“This “plant hormone” does not interfere with estrogen receptors in uterine tissue, nor with those of the breast tissue and does not induce the appearance of side effects.” Italian Newspaper*

Similarly to the Romanian article, by describing the supplement as a “plant hormone” this extract associates HS with naturally occurring substances as opposed to artificially manufactured drugs, whilst simultaneously implying credibility by invoking the language of science.

Several articles, most often in the UK, located risk less in the supplements themselves and more in user’s assumptions about them, rooting the issue more clearly in the problematic consumer information environment:

*“Do you KNOW what you’re taking? … . “herbal drugs are completely unregulated” says medical herbalist Dr [medical herbalist]. That means you may have no idea what’s really in your supplement or whether it’s right for you.” UK Newspaper*

This flags the dangers of assuming members of the public can function as competent health-consumers without access to reliable sources of information. Notably, the expert is a medical herbalist (unlikely to categorically dismiss HS) and focuses on the danger of choosing the wrong supplement rather than denouncing supplements in general.

### Financial risks

A second category of risk focused on financial rather than physical wellbeing. Several articles, most in UK newspapers, argued as their main point that supplements which had not been scientifically validated were a waste of money. Often these articles focused on recent research findings.

“According to the latest reviews, taking Echinacea regularly won't protect you from catching a cold, nor will it reduce the severity or duration. Bottom line: experts say don't waste your money.” UK Newspaper

Versions of this narrative varied greatly in the extent to which they treated lack of evidence of effectiveness as evidence of ineffectiveness. The following extract goes a step further:

*“We spend millions on them but some are a waste of money and others are BAD for you.” UK Newspaper*

This extract efficiently combines danger to both financial and physical health; suggesting users may actually be paying to make themselves ill.

### Benefits of HS as represented by the media

#### Health benefits

This theme focuses on how articles associate HS use with improvements to either general or specific aspects of wellbeing. Newspaper articles in all countries focusing on health benefits typically drew on new scientific findings about specific plants, often framed within a narrative of modern science confirming ancient wisdom:

“*People for centuries used passion-flower and kava to ease symptoms of worry and now hard-nosed researchers believe they have been right to do so.” UK Newspaper*

*“Did you know that apple cider vinegar was used once as a miracle product for weight loss and elimination of toxins? … This liquid substance for weight loss, recently rediscovered, is now available in capsule form with incredible benefits for daily life.” Romanian Newspaper*

By contrast, health benefit articles in magazines (and newspaper’s health-themed sections) rarely hinged on recent research, preferring either detailed profiles of specific plants or comparisons between different remedies for a topical health issue.

Journalists focusing on such issues would typically begin by outlining a widespread problem for which generally healthy people might nonetheless benefit from a little support from nature to strengthen their disease resistance.

*“Useful to counter the typical problems related to winter, for the welfare of the upper respiratory tract and increase the body's natural defences.” Italian Newspaper*

In such articles, the natural condition of the body is framed as a state of healthy equilibrium and illness as an externalised threat, in this case from winter colds. The role of HS here is not to heal sickness but to help the body maintain its natural wellness.

In the following example the external threat takes the form of the unnatural pace of modern living:

*“With today’s stress levels spiralling out of control, could this unassuming herb help us restore calm?” UK Magazine*

This again juxtaposes the modern and the traditional, though this time it is modern lifestyles rather than manufactured drugs being contrasted with the “unassuming” plants that “help us restore calm”, a natural redress for unnatural ills. Framing HS in relation to wellness rather than illness also suited the upbeat tone magazines preferred, allowing them to address their readers not as people with health worries but health consumers proactively managing their own wellness [15, 16].

*“Thousands of people across Europe rely on herbal medicines to improve their quality of life. They don't take them because they are sick, they take them to keep healthy.” UK Magazine*

Though most such articles framed HS as only one component of a healthy lifestyle, others positioned them as a quick and convenient way of benefiting from the healing properties of nature without necessarily having to change one’s behaviour.

#### Naturalness

This association, be it overt or implied, between a supplement’s “naturalness” and its healthiness was one of the most interesting and pervasive themes to emerge. Many articles, especially those that could not cite scientific evidence of efficacy, trumpeted a supplement’s “naturalness” as a benefit in its own right.

*“[Product] is a precious vegetal hormone which is completely natural” Italian Newspaper*

Articles varied considerably in the extent to which they explored or rationalised why naturalness was beneficial or, as in this case, vaunted it without elaboration.

It was common to associate naturalness with not just restorative powers but safety, especially in Romanian articles.

*“A completely natural treatment, aimed to both controlling the body weight and to rational, step-by-step weight reduction, without risks for general health.” Romanian Magazine*

*“Tasteless capsules are safe for the stomach. … These capsules are 100% natural and they are not medicines.” Romanian Newspaper*

The latter extract cleverly uses the dichotomy of natural HS versus manufactured medicine to make a virtue of the fact that the product may not legally be considered a “medicine”. Indeed, the ambiguous status of HS and lack of an established vocabulary for talking about them gave journalists leeway to borrow language and ideas freely from the domains of both food and medicine. At times, for example, articles’ associations+ of naturalness with healthiness echoed discourses around organic food which frame consumer self-interest in narratives driven by vague, bucolic associations rather than scientific evidence; for example the idea that organic food is good for you because it “works with nature” [63].

Unable to depict HS actively healing illness, such articles again associated “naturalness” with helping the body heal itself. They typically emphasised the supplement’s purity, intuitively linking the naturalness of its ingredients with the body’s natural defences.

*“A powerful antioxidant compound to 100% from fermented papaya, which contains substances that enhance the natural defenses against damage and oxidative stress.” Italian Newspaper*

Parle and Bansal [64] argue that HS are particularly attractive to potential users when portrayed as supporting natural healing, not healing the user but empowering them.

On the one hand this narrative is broadly consistent with the permitted role of HS as maintaining health rather than fighting illness [12]. On the other, portraying HS as less invasive than conventional drugs may again risk implying that “natural” equals “safe”, a danger emphasised by one unusually critical UK magazine article.

*“The idea of using natural herbs seems more appealing than putting chemicals into your body - but being natural doesn’t necessarily make them safe or effective.” UK Magazine*

Juxtaposing “using natural herbs” with “putting chemicals into your body” characterises one as a natural act and the other unnatural and invasive. Notably though this contrast is being used to highlight false assumptions about the relationship between naturalness and healthiness.

While it was rare for articles to declare outright that “natural” meant “safe”, journalists often conjured these notions in close enough proximity to enable the reader to make the inferential leap themselves. This particular article was unusual in deliberately stating the opposite. In this respect “naturalness” could almost be categorised as much as risk of HS as a benefit, not for what is said about it but what is left unsaid and for what readers are allowed to believe.

#### Taking responsibility for your own health

Lack of regulation and prescription allowed journalists greater latitude to present HS in a range of ways designed to appeal to their readers. Among the most prominent of these was as helping readers take responsibility for their own health maintenance. This is another sense in which the benefits of HS presented in the articles are as often about empowering users as healing them.

*“Individual choices about whether to use echinacea to treat the common cold should be guided by personal health values and preferences.” UK Newspaper*

This narrative, most prevalent in UK magazines, varied in that some articles extended it to argue that because each individual is unique no remedy will work for everyone, simultaneously excusing potential inefficacy and inviting readers to decide for themselves whether a supplement suits their needs and lifestyle. Most, though not all, articles which emphasised individual responsibility listed potential risks, framing this knowledge as essential to making an informed choice, though arguably the inclusion of risks also helps make the associated benefits seem more plausible.

While some such articles focused on the benefit of empowerment through facilitating choice, others promoted supplements based other values deemed attractive to consumers, such as fashion:

*“Take the new supplement everyone is talking about! Resveratrol, an antioxidant plant extract, is set to become the pill of 2011.” UK Magazine*

This tendency to de-emphasise a product’s original function in favour of other desirable qualities such as newness, naturalness and exclusivity when health benefit claims were prohibited echoes Jauho and Niva’s comments on functional foods [65]. Romanian newspaper articles went furthest in combining promises of health benefits with other desirable qualities.

*“Absolutely natural diet, the fastest and most famous for immediate weight loss without a prescription (for people in a hurry).” Romanian Newspaper*

These articles focused on healthiness less as a benefit in itself and more as part of a desirable lifestyle encompassing naturalness, empowerment and convenience.

### Evidence drawn upon to justify risks and benefits

#### Scientific evidence

Prominent themes included not just the types of risk and benefit claims journalists associated with HS but the types of evidence they used to justify them. New scientific findings were a preferred type of justification, though publications varied in how they used it. Newspapers, for example, were more likely to justify claims by invoking not just the name of science but also scientific language the general reader may not understand but which sounds impressive.

*“Recent studies have shown that this mechanism strengthens the intestines to defend against attacks and combat pathogens within 24 hours. The cranberry plant ingredient has an antibacterial effect and the property of preventing the adhesion of bacteria to enterocytes.” Italian Newspaper*

Such articles appeared in all countries, though citation practices varied. Italian newspapers often cited named experts from the HS industry rather than academic researchers, while Romanian articles sometimes declined to name sources.

Borrowing science’s authority but eschewing its’ imperative to critique and qualify its’ own findings helped journalists craft the sorts of unambiguous messages newspapers favour [41, 42]. Similarly, journalists enjoyed greater freedom than scientists in choosing which research to emphasise and which to ignore. New findings were often presented out of context of previous research, sometimes contradicting other recent articles without explanation; for example the following declarations appeared in same newspaper less than a month apart:

*“Echinacea does not ward off colds. The herbal remedy echinacea, which is taken to stave off colds, does not work, say leading doctors.” “Echinacea is another product known to be effective in fighting off colds and influenza-type infections.” UK Newspaper*

By contrast, magazines were less likely to justify claims with recent scientific findings than by drawing directly on expert knowledge.

#### Expertise as evidence

Magazines commonly employed expert journalists whose qualifications were treated as sufficient justification for claims of benefit or risk.

*“Three of the Best Mood-Lifting Miracles – [publication]‘s natural health expert, [name], gives us the low-down on soothing natural remedies.” UK Magazine*

Like “naturalness”, “expert” status was presented uncritically and without differentiation, rarely explaining whether their “natural health expert” is a medical doctor, nutritionist or merely a journalist specialising in health. Expert authors adopted the magazines’ customary informal tone, suggesting less a prescriptive encounter than a friendly recommendation.

*“The best approach to beating colds and flu is a holistic one – you need to develop an immunity-boosting lifestyle … my must-have supplement is [product name].” UK Magazine*

This extract also typifies the tendency of expert articles to frame HS as part of an overarching health-promoting lifestyle rather than a quick fix.

#### Tradition of use as evidence

Just as tradition of use is recognised by regulators as justifying many products’ existence, articles in all three countries cited longstanding usage as justification for featuring supplements. Rather than recommending a supplement by stating outright that tradition demonstrated its efficacy, most instead drew on the ready-made historical narratives provided by tradition of use to establish its reputation.

*“Grindelia is traditionally used in America from local populations and was introduced to Europe by missionaries” Italian Magazine*

A minority of articles emphasised both traditional and scientific justifications for benefit claims, though these were unusual.

*“Passiflora from [brand] is backed up by both tradition and using clinical and laboratory results of its calming qualities and fighting insomnia.” Romanian magazine*

More often when scientific evidence was available articles were much less likely to emphasise traditional usage as a justification.

Only a few articles elaborated on the normally implied relationship between tradition and efficacy, including one magazine piece examining assumptions underlying justification by tradition.

*Dr [Medical Herbalist] argues that traditional use is strong enough to show that herbs can be effective. “Senna has been used for generations as a laxative” he points out. “There aren’t thousands of scientific studies to back it up, but we know it works from traditional use. If it didn't, people wouldn't have been using it for 30 years or more”. UK Magazine*

Though it is clearly not the Herbalist’s intention, making this assumption explicit is a reminder that citing tradition as justifying benefit claims may be an inductive fallacy based on anecdotal evidence. It is worth remembering that whilst the implications of Traditional Herbal Remedy status are carefully defined by the EU and do not include guarantees of benefits, journalists enjoy more freedom to construe the notion of “tradition” according to their narrative goals. As with “naturalness”, it was simple for journalists to juxtapose tradition and efficacy in ways that encourage readers themselves to make the inferential leap from one to the other.

Like “naturalness”, journalists evoked tradition not just as justification in the absence of scientific evidence but as added narrative spice. Again, pro-supplement articles often framed this as a story of modern science catching up to ancient wisdom.

*For the past 4000 years, Siberian ginseng, or to give it its botanical name, Eleutheroccus senticosus, has been highly prized in Chinese medicine for increasing longevity and improving overall health … . But it's only relatively recently, when scientists started to investigate its benefits in the mid-twentieth century, that the western world has discovered the herb's secret. UK Magazine*

This exemplifies journalists’ tendency to use juxtapositions between familiar and exotic cultures, and between modern and traditional ways of knowing, to create a persuasive multi-strand narrative.

## Discussion

The dearth of reliable and authoritative information about HS has allowed lay-assumptions conflating efficacy, naturalness, tradition and safety to thrive. The gap between what consumers want to know and what conventional information sources are able to tell them creates both a challenge and an opportunity for print media, which has by default become responsible for bringing clarity to an area where very little is clear.

The thematic analysis complements findings of Entwistle and Hancock-Beaulieu’s [47] survey of health coverage in the UK press and Laboli, Caselli, Filice, Russi and Belletti’s work in Italy [66]; confirming that standards of rigor and clarity vary greatly between publications with different readerships and goals, perhaps more so than they do between countries. Though framing of benefits and risks sometimes varied nationally in terms of emphasis and citation standards, the larger narrative patterns remained remarkably consistent.

Journalists cannot invent evidence but they can choose which evidence to focus on, be it scientific, traditional or merely anecdotal. It would be naive to assume that clinical findings necessarily translate impartially into popular journalism as these are different mediums with very different remits [40]. Whereas scientific reporting is required to be dispassionate journalists are expected to engage readers’ emotions; no surprise then that they use the same findings to service disparate narrative goals.

This was particularly noticeable in newspaper articles whose authors sought to engage readers with no prior interest, for example by accentuating more controversial aspects of HS. Most magazines instead focused on the role of HS in supporting wellness, allowing them to reframe HS as a way for healthy readers to appropriate the otherwise unappealing practice of taking medicine. Such articles often framed HS as not just a valid option alongside more conventional health behaviours but an integral component of a desirable lifestyle characterised by empowerment and choice. This echoes Rayner and Easthope’s [67] description of postmodern consumer societies in which alternative medicines are prized as much for their symbolic value in identity and lifestyle construction as for health benefits. Indeed, it was as much the idea of such lifestyles these magazines were inviting readers to buy into as supplement use itself; a benefit which depends more on the symbolic resonances of HS than demonstrable efficacy.

Most articles took care to represent risks as well as benefits and avoided explicitly equating “natural” with safe, nonetheless the juxtaposition of the romanticised concept of “naturalness” with the concrete issue of health outcomes remained a problematic one [21]. Knight [68] finds that popular writing about food increasingly conflates the concepts “natural”, “healthy” and “good”, often setting them against an opposing triad of “artificial,” “toxic” and “evil”. Knight traces this association back to an idealised natural state of man, uncorrupted by modern society, similar to that romanticised by Jean Jacques Rousseau. When combined with discourses of traditional use, this frames HS as a way of recalling an earlier state of natural healthiness and moral virtue.

The exact status of traditional use in relation to other kinds of justifying evidence is debatable, though Helmstädter and Staiger’s [69] proposed hierarchy of evidence types locates well-established “therapeutic wisdom” somewhere between scientific findings and the opinions of individual experts [70]. This has the advantage of offering a potential means of stratifying the three types of evidence discussed above.

The individual experts who contributed to the articles surveyed in this study typically adopted the role of a friendly guide to a potentially confusing realm, echoing a magazine editor’s description in Doel and Segrott [44] of “a sort of translator between experts who can often talk in quite impenetrable jargon, and just normal everyday people like us”, (p. 140). This cleverly mimics the tendency for HS users to learn about supplements via recommendations from friends rather than health practitioners [22].

It remains unclear whether the unique information environment surrounding HS genuinely empowers consumers in the manner described by Koinig et al. [17]. Crucially though, the HS consumer is also a consumer of media. They decide not just which supplements to use but which publications to buy; it is therefore ultimately their perceived interests that journalists tailor their articles towards.

The regulatory confusion surrounding HS was referred to by only a few articles, usually when it could be related directly to the aforementioned perceived interests. Accordingly, when it was referred to it was most often as a way to support narratives depicting these products as being less intrinsically safe and controlled than consumers might assume, with only the one UK newspaper article cited above delving more deeply. Articles were even less likely to describe the inconsistency of regulations between countries, focusing instead on stories judged to appeal to their specific readership in their own country of publication. Nor did the specific regulatory nuances found in different countries appear be clearly reflected in how journalists in those countries wrote about HS. Instead the relevance of the regulatory inconsistency and confusion was that it stifled other channels of communication that more directly reflect these various approaches to regulation, leaving the print media both relatively unaffected and largely unopposed as a dominant voice in the consumer information environment. This highlights a crucial need for more articles which raise awareness of the regulatory confusion that makes communicating HS information so problematic.

The freedom afforded print media allows a wide enough range of perspectives that consumers are empowered to choose not just which supplements but also which messages to consume. The risk lies in them only reading articles telling them what they want to hear rather than alternative views that might enable more balanced decisions. Allowing print media to be a primary source of information for HS users therefore provides no means of ensuring that potential users will happen across the information that will enable them to make the best choice.

This highlights a central paradox of the HS marketplace, namely that consumers are encouraged to make their own decisions about their healthcare [13] but may never receive the information which enables them to do so effectively. Is an empowered consumer simply one who chooses for themselves what to consume or must they also be equipped with the knowledge required to make those choices meaningful?

The study has some limitations, notably a relatively small sample size and the difficulties of translating sometimes subtle and complex messages from one language to another. Integrating qualitative research from more than one country is likewise an inherently cumbersome process [60]. Furthermore it only captures articles published during a limited period and, taking print articles as its sole source of data, is largely unable to gauge the effects of the inconsistent ways in which these products are regulated on consumers.

## Conclusion

Print media’s dominance of the HS consumer information environment is ultimately an accident: the result of other voices being stifled, leaving theirs the loudest in the room. Most newspapers and magazines do not exist chiefly to disseminate public health information [66] so they cannot be condemned for failing in a role they were never intended to fill.

Clearly it is not enough to remove all HS information with the potential to mislead. Its place must be taken by impartial, accessible and trustworthy information lest the resulting knowledge vacuum become a breeding ground for dangerous fallacies, such as the assumption that “natural” always means safe.

Until consumers have reliable access to a source of such information the danger remains that HS users will be allowed to believe that supplements offer them a panacea as safe as food and as efficacious as medicine.

Though a relatively small-scale qualitative study that looks at how risks and benefits associated with HS are described and justified in print media, this work might be useful in helping to design larger scale, longer term studies of the consequences of the confusion and inconsistency that continues to plague the supplement industry. A useful approach, for example, would be a longitudinal study spanning at least 5 years, perhaps using content analysis, to provide a rigorous analysis of how products classified in different ways in different countries are depicted in different types of media at different points in time.

Future research in the area might also focus specifically on how consumers use online sources of information about HS, how readers of newspapers and magazines interpret and act on attributions of “naturalness” and “tradition” and how this interacts with existing beliefs and word of mouth information, or the decision-making processes of consumers choosing between HS and other healthcare options.

## Data Availability

The datasets used and/or analysed during the current study are available from the corresponding author on reasonable request.

## Abbreviations

CAM: Complementary and Alternative Medicine
HS: Herbal Supplements
MHRA: Medicine and Healthcare products Regulatory Agency
UK: United Kingdom

## Footnotes

1 HS are generally implied to fall within the legal definition of Food Supplements (EU Directive (2002/46/EC), though this is not stated as specifically as might be wished. Therefore it was deemed important to select a consistent term which could be precisely defined and adhered to irrespective of the inconsistent terminology and classification found at the level of Individual EU states. Thus, the definition of HS decided upon was: “foodstuffs the purpose of which is to supplement the normal diet and which are concentrated sources of botanical preparations that have nutritional or physiological effect, alone or in combination with vitamins, minerals and other substances which are not plant-based. HS are marketed in dose form, such as capsules, pastilles, tablets, pills and other similar forms, sachets of powder, ampoules of liquids, drop dispensing bottles, and other similar forms of liquids and powders designed to be taken in measured small unit quantities”. This definition is reflected in other publications drawn from the PlantLIBRA project already in print, though the project tended to prefer the term “Plant Food Supplements” to “Herbal Supplements” to refer to the same products, drawing in particular from survey published by Garcia-Alvarez [48].
